# Application of Telemedicine in COVID-19: A Bibliometric Analysis

**DOI:** 10.3389/fpubh.2022.908756

**Published:** 2022-05-26

**Authors:** Xue Lan, Han Yu, Lei Cui

**Affiliations:** School of Health Management, China Medical University, Shenyang, China

**Keywords:** telemedicine, COVID-19, eHealth, bibliometric analysis, network analysis

## Abstract

**Background:**

Telemedicine as a tool that can reduce potential disease spread and fill a gap in healthcare has been increasingly applied during the COVID-19 pandemic. Many studies have summarized telemedicine's technologies or the diseases' applications. However, these studies were reviewed separately. There is a lack of a comprehensive overview of the telemedicine technologies, application areas, and medical service types.

**Objective:**

We aimed to investigate the research direction of telemedicine at COVID-19 and to clarify what kind of telemedicine technology is used in what diseases, and what medical services are provided by telemedicine.

**Methods:**

Publications addressing telemedicine in COVID-19 were retrieved from the PubMed database. To extract bibliographic information and do a bi-clustering analysis, we used Bicomb and gCLUTO. The co-occurrence networks of diseases, technology, and healthcare services were then constructed and shown using R-studio and the Gephi tool.

**Results:**

We retrieved 5,224 research papers on telemedicine at COVID-19 distributed among 1460 journals. Most articles were published in *the Journal of Medical Internet Research* (166/5,224, 3.18%). The United States published the most articles on telemedicine. The research clusters comprised 6 clusters, which refer to mental health, mhealth, cross-infection control, and self-management of diseases. The network analysis revealed a triple relation with diseases, technologies, and health care services with 303 nodes and 5,664 edges. The entity “delivery of health care” was the node with the highest betweenness centrality at 6,787.79, followed by “remote consultation” (4,395.76) and “infection control” (3,700.50).

**Conclusions:**

The results of this study highlight widely use of telemedicine during COVID-19. Most studies relate to the delivery of health care and mental health services. Technologies were primarily *via* mobile devices to deliver health care, remote consultation, control infection, and contact tracing. The study assists researchers in comprehending the knowledge structure in this sector, enabling them to discover critical topics and choose the best match for their survey work.

## Introduction

The COVID-19 pandemic, which began as a case of pneumonia, has evolved into a worldwide pandemic. Numerous precautions have been adopted to slow the spread of illness and minimize the danger of infection, including personal protective equipment, social isolation, careful contact tracing and quarantine, and lockdown limitations. In this environment, novel technologies are required to improve the efficiency and quality of health care while also reducing the danger of transmission. Telemedicine is well-positioned to meet these demands *via* web-based, mobile phone-based, and virtual reality-based screening, diagnosis, and self-management of health (1). The World Health Organization defines telemedicine as “the delivery of health care services, where distance is a factor, by all health care professionals using information and communication technologies for the exchange of valid information for the diagnosis, treatment, and prevention of disease and injury, research and evaluation, and continuing education of health care providers, all to advance individual and population health” (2). When it comes to delivering patient care, telemedicine has emerged as an indispensable and successful choice.

As the epidemic looms worldwide, telemedicine is indisputably vital for the worldwide management of COVID-19. Leveraging telemedicine for COVID-19 allows patients to be efficiently screened before they arrive in the emergency department. As a result, patients, physicians, and public members are protected from exposure. It is patient-centered as well as favorable to self-quarantine. In the early months of 2020, as the number of COVID-19 patients grew, so did the number of online searches for telehealth and telemedicine in the United States (3). Telehealth applications, acceptability, and use cases in the ambulatory setting have grown as a consequence of the epidemic in nearly every discipline (4, 5). Aside from independent practitioners' examination and administration, telemedicine was widely used to provide a broad spectrum of consultation (6), diagnosis (7), symptom monitoring (8), contact tracing (9), and psychological intervention (10). Telemedicine applications based on a variety of information technologies are also becoming more common (11), like artificial intelligence (12), robotic technologies (13), and wearable metabolic biosensors (14). The great potential shown by telemedicine has received attention from researchers in many countries (15). Understanding the technologies used for health care services in COVID-19 can enable a better understanding of the telemedicine implementation in COVID-19.

Researchers in these fields produced several valuable meta-analyses and systematic reviews to investigate the current state of telemedicine research. For example, Bokolo (11) discusses telemedicine and eHealth as a proactive technique to enhance clinical care using data from the available literature. The results of this research demonstrate the importance of telemedicine and the existing applications used during the epidemic. Bahl et al. (16) review the technologies used for the COVID-19 pandemic and its significant benefits. Bokolo (17) reviewed 35 research publications published between January 2019 and May 2020 to discuss the significance of telemedicine and virtual care for distant patient treatment. These studies investigated specific telemedicine applications and related technologies.

Other relevant systematic reviews have explicitly focused on the effectiveness of telemedicine in heart failure (18), headache (19), chronic pain (20), and other diseases. These reviews and meta-analyses have improved our understanding of telemedicine. However, these studies summarize the telemedicine applications in a particular illness or technologies used for telemedicine, lacking an analysis in an integrated way. Due to the limited number of review papers and researchers' perspectives regarding the focus of each review, we lack a comprehensive, big-picture overview of the telemedicine trends and issues that have been the focus of recent empirical investigations. Publication bias, search bias, and selection bias are common methodological problems and information gaps in literature studies (21). The study given in this paper uses bibliometric analysis, a quantitative approach, to solve these issues.

Bibliometric analysis has been extensively utilized to characterize the hot areas and contributions of researchers, journals, and countries/regions in a complete review of academic literature (22, 23). Quantified numerical data may also aid researchers in predicting future trends (24). In the current study, we used bi-clustering analysis to simultaneously realize the co-citation and bibliographic coupling analyses. The two statistical methods in bibliometrics—co-citation analysis and bibliographic coupling analysis —were often used to explore the structure and frontiers of research in the field (25, 26). The difference is that the co-citation analysis focuses on the cited literature, which demonstrates the disciplinary knowledge base of the area. In contrast, the bibliographic coupling analysis focuses on citing literature, representing the discipline's frontiers of research. Both methods have been studied separately and without integrated analysis. The bi-clustering approach combines the strength of the two ways to reveal the foundation and frontiers of research (27). Moreover, we used network visualization to describe the technology used in telemedicine and its connections with diseases and health care services.

This article attempts to provide a general description of quantitative and visual information in the literature on telemedicine research at COVID-19, identifying emerging trends and potential hot spots from various aspects, including diseases, health services, and technologies. This study was conducted to address the following questions: (1) What are the hot spots and trends in telemedicine? (2) What kind of technologies are used in telemedicine? and (3) What are the diseases provided medical services by telemedicine, and how may telemedicine be utilized to treat them?

## Methods

### Research Design

**Figure 1:**
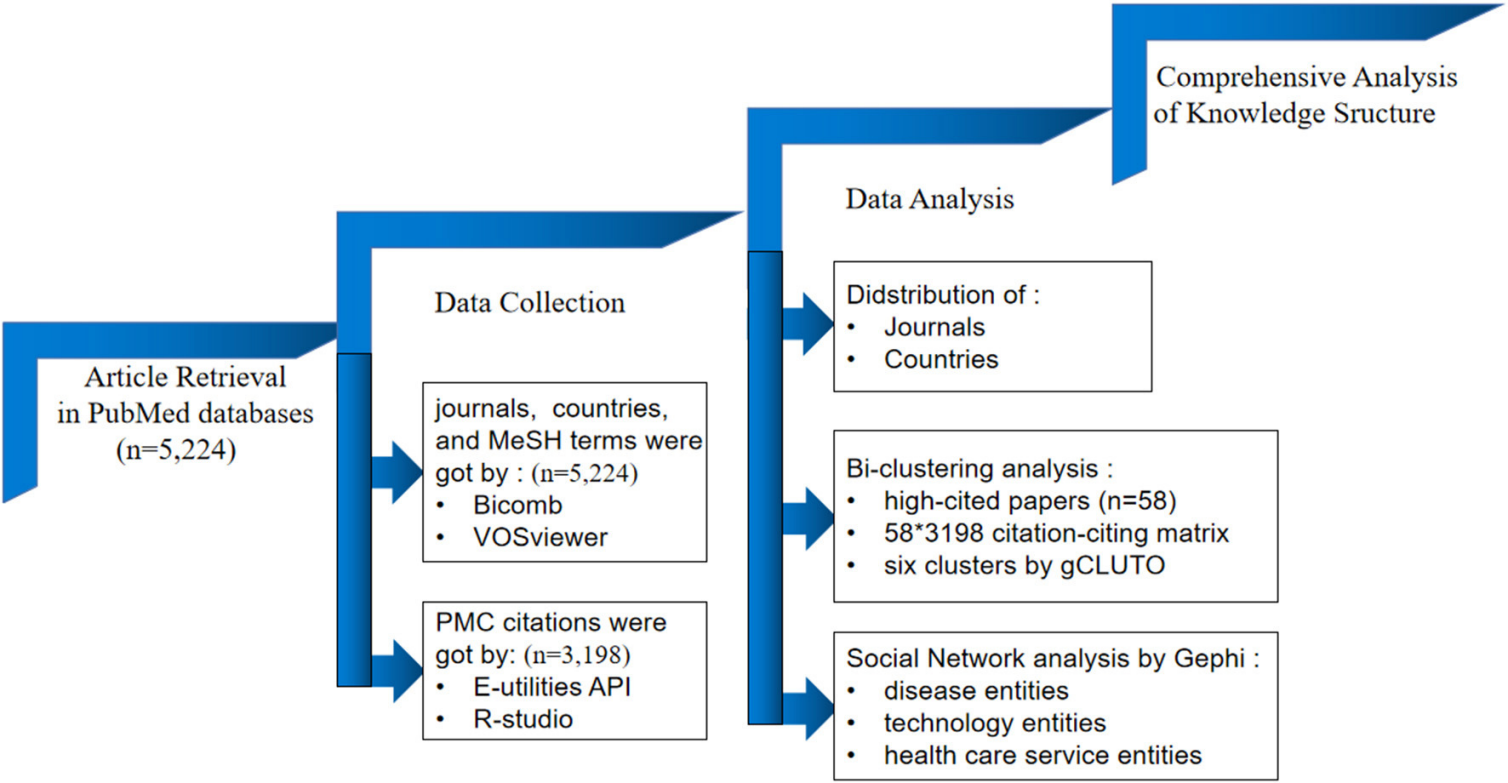
Research design framework.

This study was organized using a research design framework (Figure 1). Specifically, we completed the following sequence of steps: (1) retrieving relevant research articles during COVID-19 with the search term telemedicine; (2) collecting publication data in the PubMed database and getting the articles with PMC citations; (3) identifying descriptive information, such as distribution of publications by journals, countries, as well as an illustration of top cited articles by VOSviewer; (4) applying bi-clustering analyses of the citation-citing matrix to get the research topics with the help of gCLUTO software; (5) selecting the terms of disease, technology, and health care service; (6) constructing the network through the co-occurrence of three type nodes by Gephi.

### Information Sources

PubMed was used to collect data for the following reasons: (1) Publications in PubMed are indexed using MeSH (Medical Subject Headings) terminology, a collection of standardized phrases that may describe the content of articles. MeSH phrases are structured hierarchically, and MeSH descriptors indicate several semantic kinds (i.e., disease and chemicals). (2) Entrez Programming Utilities (E-utilities) was provided to develop particular queries on PubMed. This function can retrieve the citation relation of an article in PubMed. The acquired citation dataset can be enriched with the MeSH terms.

This research gathered relevant papers using an unrestricted PubMed search. The actual search string used was: (“telemedicine”[Mesh] OR “mobile applications”[Mesh] OR “smartphone”[Mesh]) AND (COVID-19[Mesh] OR SARS-CoV-2[MESH]), which was retrieved on December 16, 2021. The titles, authors, countries, publication year, and MeSH terms were downloaded and stored in PubMed format.

### Data Collection and Bi-clustering Analysis

R-studio software was used to collect the citation relation of the citing papers by e-Link functions and to develop the matrix of high-cited papers and citing papers for the bi-clustering analysis.

The bi-clustering analysis was performed by gCLUTO software to simultaneously realize the co-citation analysis and bibliographic coupling analysis. The bi-clustering options were as follows: Method: repeated bisection; the number of clusters: 6; similarity function: cosine; criterion function: I2; the number of iterations: 10.

### Interpretation of the Clustering Results

We used the MAJR (MeSH Major Topic) to summarize the themes of each category by the TF-IDF method. Due to the fact that not all words in a document are equal in significance of each term was determined by multiplying term frequencies (TF) by the inverse document frequency (IDF) for that term. The equation represents the procedure for calculating TF-IDF.


$$\begin{array}{l} TF-IDF\left(Term_{a}\right)=TF_{a}*\log\left(\frac{N}{n_{a}}\right) \end{array}$$

Where TFa denotes the number of instances of the word A in the current class, N is the total number of clusters, and n_a_ indicates the number of clusters containing the term A. This study was conducted to address RQ1.

### Social Network Analysis of Entities

We used the MeSH tree structure to distinguish distinct semantic types to solve the problem. Each MeSH term has at least one MeSH tree number. A MeSH tree is divided into 16 categories: anatomical words are classified as category A, biological phrases are classified as category B, illness terms are classified as category C, etc. A MeSH tree structure enables the qualification of MeSH concepts belonging to separate semantic kinds using particular descriptors and numbers (28). For example, the MeSH terms of telemedicine can be qualified by “L01.178.847.652”. To discover the technology used for health care services, the entities of technology, diseases, and health care services were extracted based on MeSH tree numbers “C/F01/F03,” “L,” and “N” by R-studio.

Finally, we used network analysis and visualization to ascertain the relationships between the various MeSH term types. The Gephi software was employed to construct the network. The network nodes are the entities of technology, diseases, and health care services, and the links represent the co-occurrence frequency of these entities. To understand the connections between these entities, we calculate the betweenness centrality of each node. The betweenness centrality, which is derived by the frequency with which a node intersects the geodesic trajectories of other network nodes, indicates a node's impact in a network (29). This analysis was performed to answer RQ2 and RQ3.

## Results

### Publishing Outputs

We identified and incorporated 5,224 publications on telemedicine in COVID-19 from PubMed based on our search strategy. Literature related to telemedicine in COVID-19 was distributed among 1,460 journals; 650 have published only one paper on mobile health apps. The top 10 journals are shown in Table 1. *The* Journal of Medical Internet Research was the most productive publication source (166/5,224, 3.18%), followed by *Telemedicine Journal and e-health* (118/5,224, 2.26%), then *International Journal of Environmental Research and Public Health* (93/5,224, 1.78%). And then used the 2020 Journal Citation Reports (JCR) to search the Impact Factor (IF) of the top 10 journals. The highest IF belonged to the Journal of Medical Internet Research (5.428), followed by *JMIR mHealth and uHealth* (4.773).

**Table 1:** Top 10 journals are publishing research on telemedicine research in COVID-19.

**Rank**	**Journal**	**Country**	**IF**	**Publications**
1	Journal of Medical Internet Research	Canada	5.428	166
2	Telemedicine Journal and e-health	United States	1.297	118
3	International Journal of Environmental Research and Public Health	Nigeria	3.390	93
4	PLoS One	United States	3.240	64
5	JMIR mHealth and uHealth	Canada	4.773	58
6	Journal of Diabetes Science and Technology	United States	None	43
7	Journal of the American Medical Informatics Association	England	4.497	43
8	Indian Journal of Ophthalmology	India	1.848	41
9	Asian Journal of Psychiatry	Netherlands	3.543	37
10	Frontiers in Public Health	Switzerland	3.709	37

### Distribution of Countries

**Figure 2:**
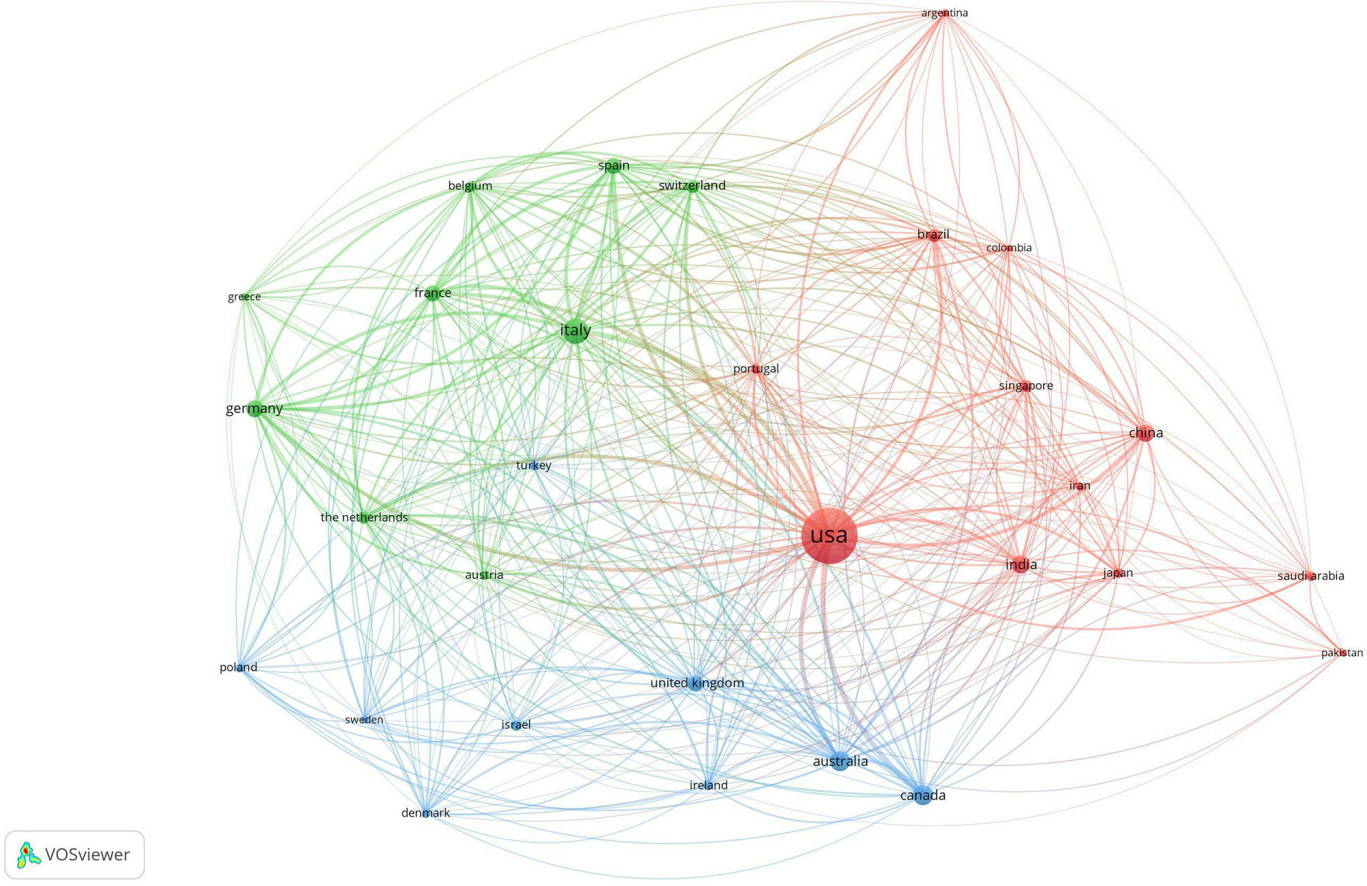
The cooperation of countries/regions in telemedicine at COVID-19.

Between 2019 and 2021, a total of 30 nations published more than 30 publications in telemedicine research. The United States published the most articles (*n* = 1,977); Italy (*n* = 387) and Australia (*n* = 243) followed. The United States publishes the most articles and collaborates most with the other nations. VOSviewer was used to cluster the data and visualize it, as seen in Figure 2. These 30 countries were divided into 3 clusters: the first cluster (represented by the red node) contained 12 countries led by the United States; the second cluster (represented by the green node) contained 9 countries led by Italy and Germany, and the third cluster (represented by the blue node) contained 9 countries led by Australia and Canada.

### High-Cited Papers

The query retrieved 5,224 articles in PubMed, and 3,198 articles have PMC citations. The top 10 cited references are shown in Table 2. The cited times in Table 2 correspond to the number of citations in the literature that we retrieved. The publication that received the most citations, Dr. Judd E. Hollander's “Virtually Perfect? Telemedicine for COVID-19,” was published in the *New England Journal of Medicine* in 2020. All of the top 10 references were published in 2020. The top 10 cited references concentrate on the clinical characteristics of COVID-19 patients and the use of telemedicine during COVID-19.

**Table 2:** Top 10 cited references in telemedicine research in COVID-19.

**PMID**	**Title**	**Year**	**Cited Times**	**Journal**	**IF**
32160451	Virtually Perfect? Telemedicine for COVID-19	2020	314	New England Journal of Medicine	91.253
31986264	Clinical features of patients infected with 2019 novel coronavirus in Wuhan, China	2020	121	Lancet	79.323
32196391	telemedicine for global emergencies: Implications for coronavirus disease 2019 (COVID-19)	2020	109	Journal of Telemedicine and Telecare	6.184
32109013	Clinical Characteristics of Coronavirus Disease 2019 in China	2020	106	New England Journal of Medicine	91.253
32091533	Characteristics of and Important Lessons From the Coronavirus Disease 2019 (COVID-19) Outbreak in China: Summary of a Report of 72 314 Cases From the Chinese Center for Disease Control and Prevention	2020	102	JAMA-Journal of The American Medical Association	56.274
32165352	Video consultations for COVID-19	2020	93	BMJ-British Medical Journal	39.89
32171076	Clinical course and risk factors for mortality of adult inpatients with COVID-19 in Wuhan, China: a retrospective cohort study	2020	86	Lancet	79.323
32324855	COVID-19 transforms health care through telemedicine: Evidence from the field	2020	84	Journal of the American Medical Informatics Association	4.497
32031570	Clinical Characteristics of 138 Hospitalized Patients With 2019 Novel Coronavirus-Infected Pneumonia in Wuhan, China	2020	81	JAMA-Journal of the American Medical Association	56.274
32066541	Cancer patients in SARS-CoV-2 infection: a nationwide analysis in China	2020	81	Lancet Oncology	41.316

### Bi-clustering Analysis

**Figure 3:**
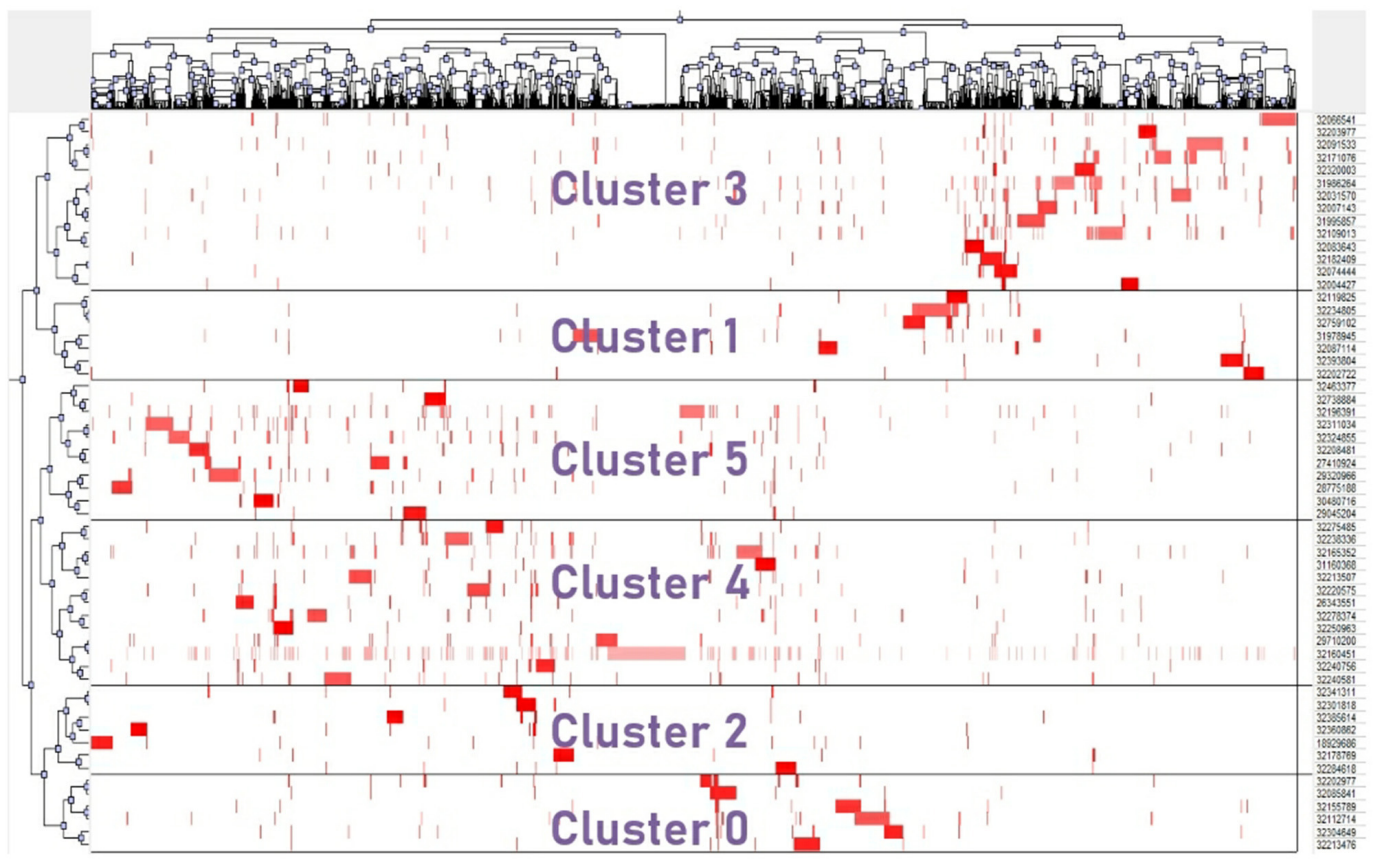
Matrix visualization of bi-clustering of the cited-citing matrix.

We generated a 58^*^3,198 citation-citing matrix using the top 58 highly cited references that were referenced at least 30 times in the retrieved publications. R-studio created the matrix in order to facilitate future bi-clustering study. The bi-clustering findings revealed six major clusters, as seen in Figure 3; each cluster had associated row and column subsets, each of which represented a local mode with a high degree of similarity between highly cited articles and papers cited by co-occurrence.

To summarize the research themes of each cluster, the TF-IDF method was used to extract the significant MAJR terms. The top 10 MAJR in each cluster is given in Table 3. These six categories may reflect the primary telemedicine research issues in COVID-19.

(1) Cluster 0: telemedicine in the therapy of mental health during COVID-19;
(2) Cluster 1: the usefulness of mobile health devices in COVID-19 prevention and monitoring of close contacts;
(3) Cluster 2: telemedicine ensures patient access to care;
(4) Cluster 3: usage of telemedicine technology to prevent cross-infection and to monitor patient medication during isolation;
(5) Cluster 4: application of telemedicine technologies to treat chronic diseases;
(6) Cluster 5: The role of electronic health records and clinical decision support systems during COVID-19.

**Table 3:** Top 10 TF-IDF values of MAJR in each cluster.

**Cluster**	**Rank**	**MAJR**	**TF-IDF**
0	1	Mental disorders/epidemiology	0.002010
	2	Mental disorders/psychology	0.001608
	3	Mental health	0.001335
	4	Crisis intervention/methods	0.001311
	5	Psychotic disorders/therapy	0.001311
	6	Mental health/trends	0.001311
	7	Behavior therapy/standards	0.001311
	8	Mental disorders/prevention and control	0.001311
	9	Adaptation, psychological	0.001268
	10	Social support	0.001206
1	1	Contact tracing/ethics	0.003682
	2	Contact tracing/instrumentation	0.002455
	3	Contact tracing	0.002137
	4	Privacy	0.001899
	5	Contact tracing/statistics and numerical data	0.001841
	6	Mobile applications/statistics and numerical data	0.001528
	7	Disease notification/methods	0.001227
	8	Pandemics/ethics	0.001227
	9	Confidentiality/ethics	0.001227
	10	Population surveillance/methods	0.001227
2	1	Technology	0.001924
	2	Visual acuity	0.001569
	3	Abortion, induced/methods	0.001569
	4	Orthopedic procedures	0.001443
	5	Orthopedics/methods	0.001214
	6	Eye diseases/diagnosis	0.001065
	7	Artificial intelligence/trends	0.000962
	8	Traumatology/methods	0.000962
	9	Ophthalmology/organization and administration	0.000962
	10	Reconstructive surgical procedures/methods	0.000962
3	1	Cross infection/prevention and control	0.000925
	2	Antineoplastic agents/therapeutic use	0.000755
	3	Defibrillators, implantable	0.000755
	4	Clinical trials as topic	0.000755
	5	Inflammatory bowel diseases/epidemiology	0.000755
	6	Coronavirus infections/immunology	0.000755
	7	Hospitalization/statistics and numerical data	0.000617
	8	Neoplasms/radiotherapy	0.000617
	9	Infection control/standards	0.000584
	10	Monitoring, physiologic/methods	0.000512
4	1	Primary health care/methods	0.000903
	2	Endovascular procedures/methods	0.000700
	3	Kidney failure, chronic/therapy	0.000700
	4	Neurosurgical procedures/methods	0.000700
	5	Dermatology/education	0.000700
	6	Primary health care/trends	0.000700
	7	Hypersensitivity/therapy	0.000572
	8	Skin diseases/diagnosis	0.000572
	9	Inflammatory bowel diseases/therapy	0.000572
	10	Remote consultation/organization and administration	0.000542
5	1	Physical therapists	0.778151
	2	Health services	0.778151
	3	Urologic diseases/epidemiology	0.778151
	4	Workflow	0.778151
	5	Academic medical centers	0.778151
	6	Medical informatics applications	0.477121
	7	Physician-patient relations	0.477121
	8	Urology	0.301030
	9	Chronic pain/therapy	0.477121
	10	General practice	0.477121

### Telemedicine During COVID-19 Entities Network Analysis

This study considered three entity types: technology, disease, and health care service. The results of the named entity extraction in MeSH terms are shown in Table 4. With 205 Mesh words, the most often occurring entity category was health care service. Some terms have more than one tree number. For example, “telepathology” is both in L and N categories. To distinguish them, we manually categorized some words. Besides, some terms have very similar meanings, such as the terms “cell phone,” “smartphone,” and “telephone”. In this study, we unified them as “telephone”. Finally, we got 303 entities of three node types.

**Table 4:** Entity extraction results.

**Type**	**Mesh terms**	**Tree numbers**	**Entity counts**
**Technology**			
	Information science category	L	30
**Disease**			
	Diseases category	C	75
	Behavior and behavior mechanisms	F01	
	Mental disorders	F03	
**Health care service**			
	Delivery of health care	N04.590.374	205
	Health services	N02.421	
	Public health	N06.850	

The relation extraction procedure was used to determine the co-occurrences of various entities inside a single document. After extracting the entities and relations, we used them in the network analysis to ascertain the degree of each entity (node) and connection (edge). The network is an indirect graph that integrates the related research on telemedicine in COVID-19 in a total of 303 nodes and 5,664 edges. The network was evaluated using betweenness centrality. Among the 303 nodes, the entity “delivery of health care” was the most significant node with a betweenness centrality at 6,787.79, followed by “remote consultation” (4,395.76), “infection control” (3,700.50), “mobile applications” (3,390.40), and “health services accessibility” (3,009.27). The “delivery of health care” node had a strong influence on the disease nodes, including “neoplasms” and “mental disorders.” In addition, it had a strong connection with the technology nodes, “mobile applications,” “videoconferencing,” and “virtual reality.”

The findings showed that “mobile applications,” “telephone,” “videoconferencing,” “internet,” “social media,” and “remote sensing technology” were significant technological nodes. Additionally, the entities of emerging diagnostic and therapeutic technologies, such as “artificial intelligence,” “virtual reality,” and “deep learning,” were shown. Artificial intelligence was shown to be strongly associated with “eye disorders,” “heart diseases,” and “urogenital neoplasms.” Artificial intelligence was first used to give remote consultation and to track down connections.

**Figure 4:**
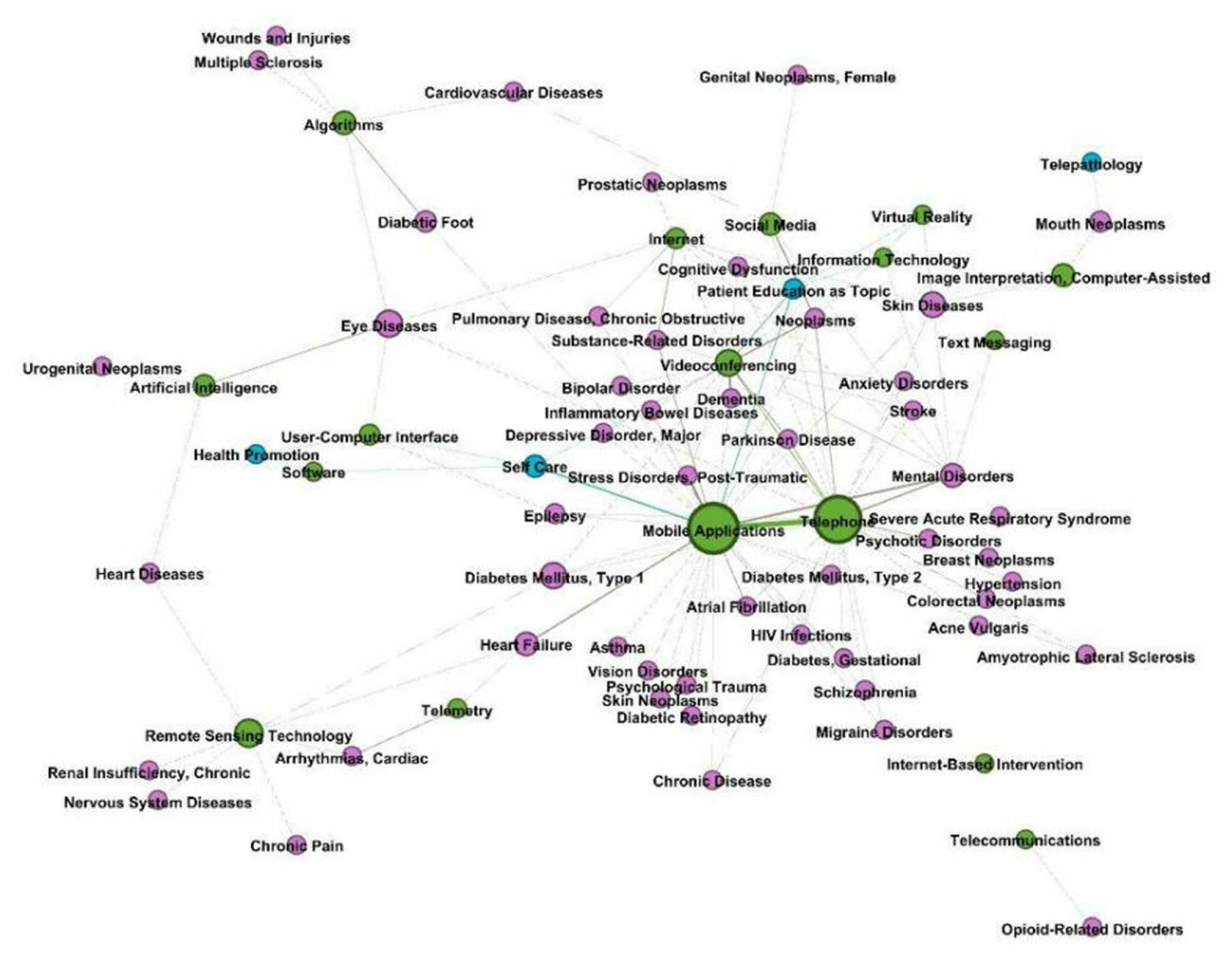
Technology entity nodes and other node connections in the network.

Figure 4 illustrates numerous linkages between nodes representing technological entities and other nodes. The analysis revealed that the node “mobile apps” was more closely related to chronic illness entities such as type 1/type 2 diabetes, heart failure, and asthma, as well as with health service entities (patient education and self-care). Additionally, the node “telephone” was more closely related with entities associated with mental diseases, such as anxiety disorder, than it was with the technological entity type itself (mobile applications, video conferencing, and social media).

### Common Diseases With Technology Entities Network

We intended to research the most prevalent disorders and determine the primary telemedicine technologies employed in this procedure. After selecting the most frequently detected illness entities in the dataset, the relation extraction findings were filtered. The figures in parentheses in this report represent the prevalence of each illness discovered in the PubMed dataset. “Neoplasms” (167), “mental disorders” (130), “diabetes mellitus” (84), “skin diseases” (57), “opioid-related illnesses” (51), “epilepsy” (49), “diabetes mellitus, type 1” (48), “cardiovascular diseases” (44), “stroke” (39), and “heart failure” (38).

**Figure 5:**
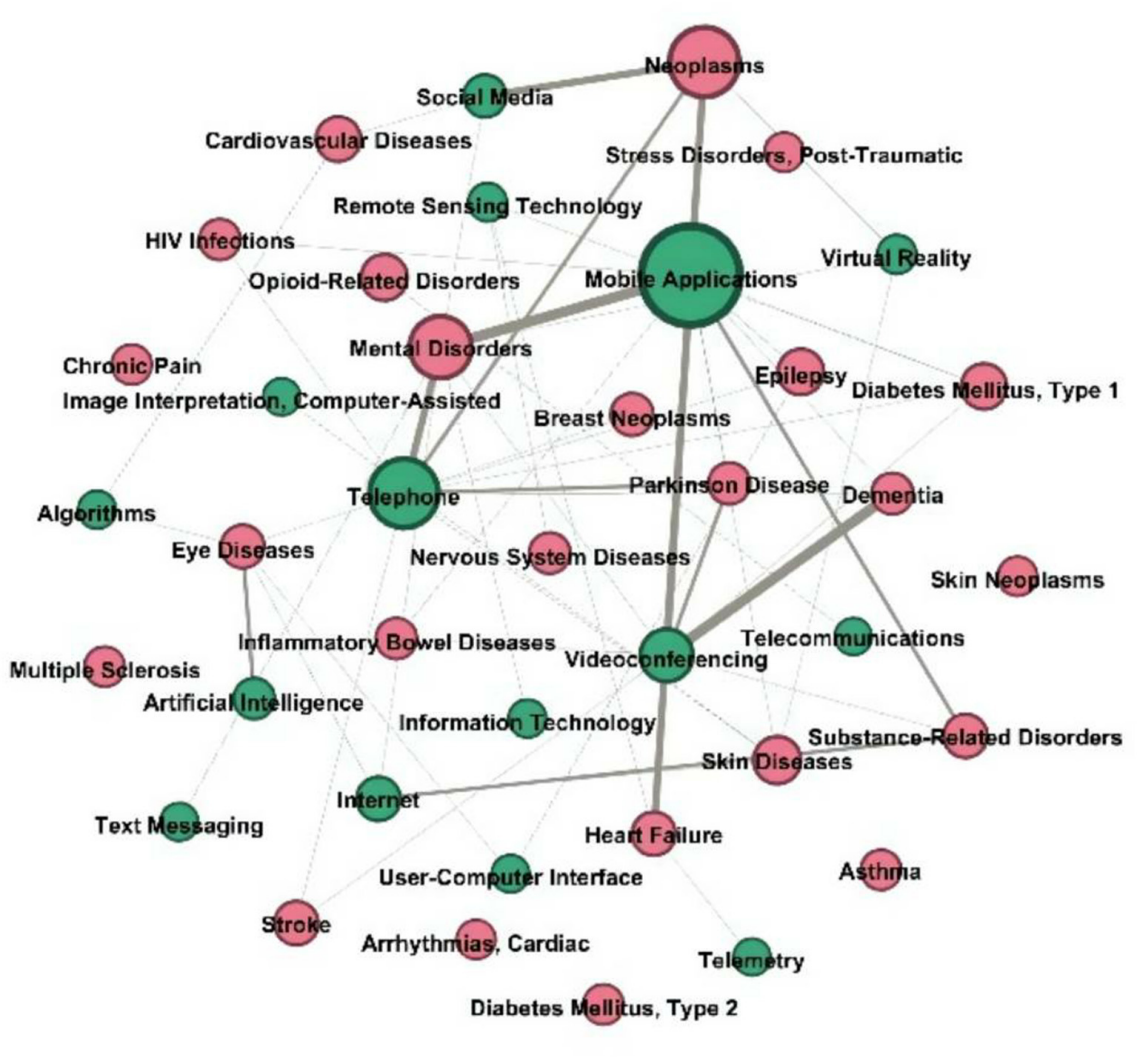
Common diseases with IT entities network.

For the network study, only the top 30 most prevalent illnesses and their relationships remained (Figure 5). According to network analysis, the two most significant technological entities associated with telemedicine were “mobile apps” and “telephone” (weighted degree of 11). Additionally, the dataset included additional IT phrases related to the top disorders. These included the “internet” (which is connected with mental illnesses, eye problems, skin diseases, and drug use disorders) and “remote sensing technologies” (associated with nervous system diseases, heart failure, and type 1 diabetes mellitus).

### Entity Association in a Specific Disease Network

We explored the entities and relationships of the top three most prevalent illnesses in the telemedicine research field: “neoplasms,” “mental disorders,” and “diabetes mellitus.” This investigation examined the technology used to diagnose and treat ailments, as well as the delivery of health care.

**Figure 6:**
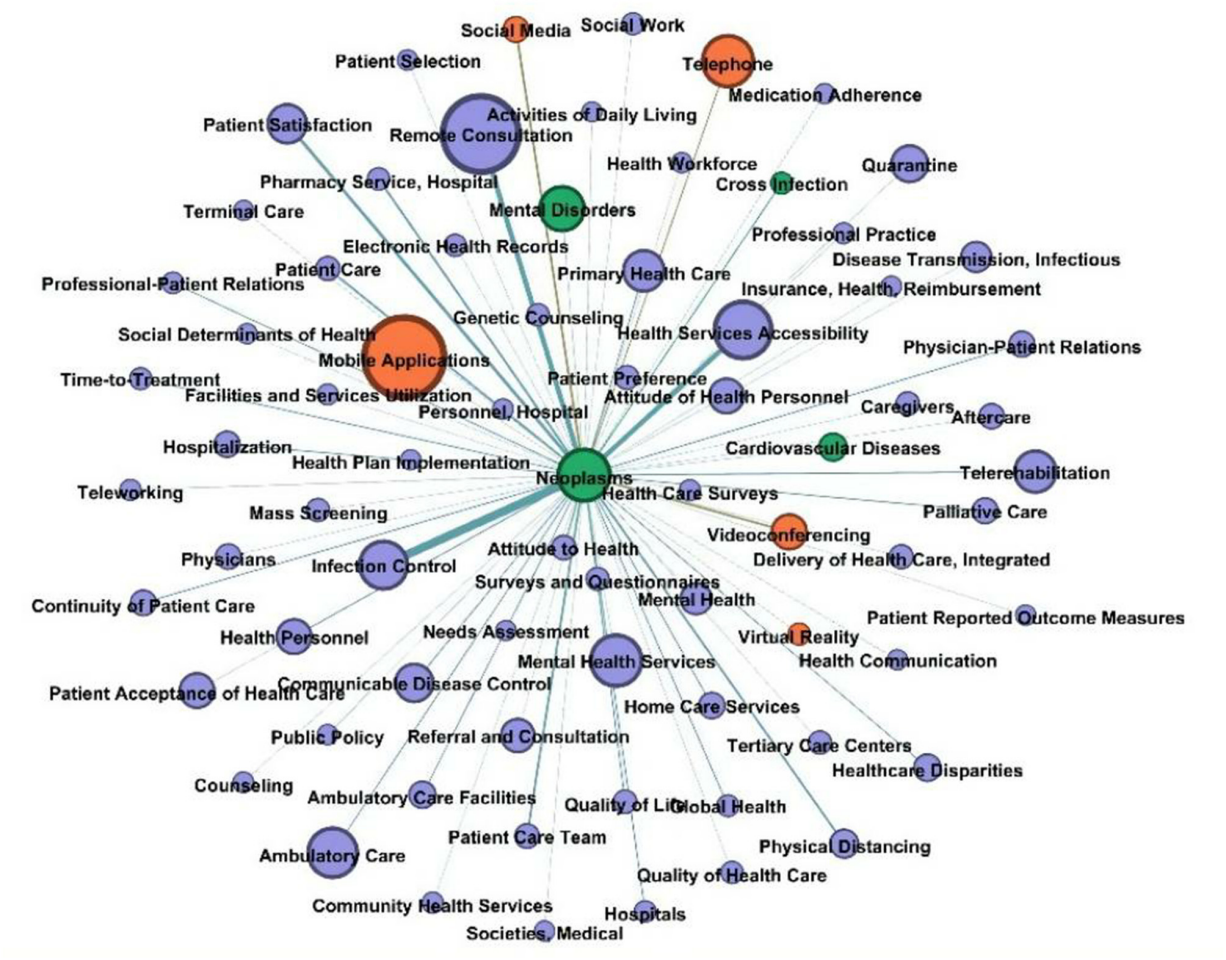
Neoplasm node and other nodes connections in the network.

Figure 6 displays the neoplasms network, where signs of “infection control” occurred most frequently for this disease. Many scholars are interested in using “remote consultation” to reduce the likelihood of infection in all patients with cancer. Besides, the other essential entities were “patient satisfaction,” “health services accessibility,” “professional-patient relations,” and “patient care team.” The significant technology entities were “videoconferencing,” “mobile applications,” “telephone,” “virtual reality,” and “social media.” The study focuses on remote consultation, infection control, and the patient access experience.

**Figure 7:**
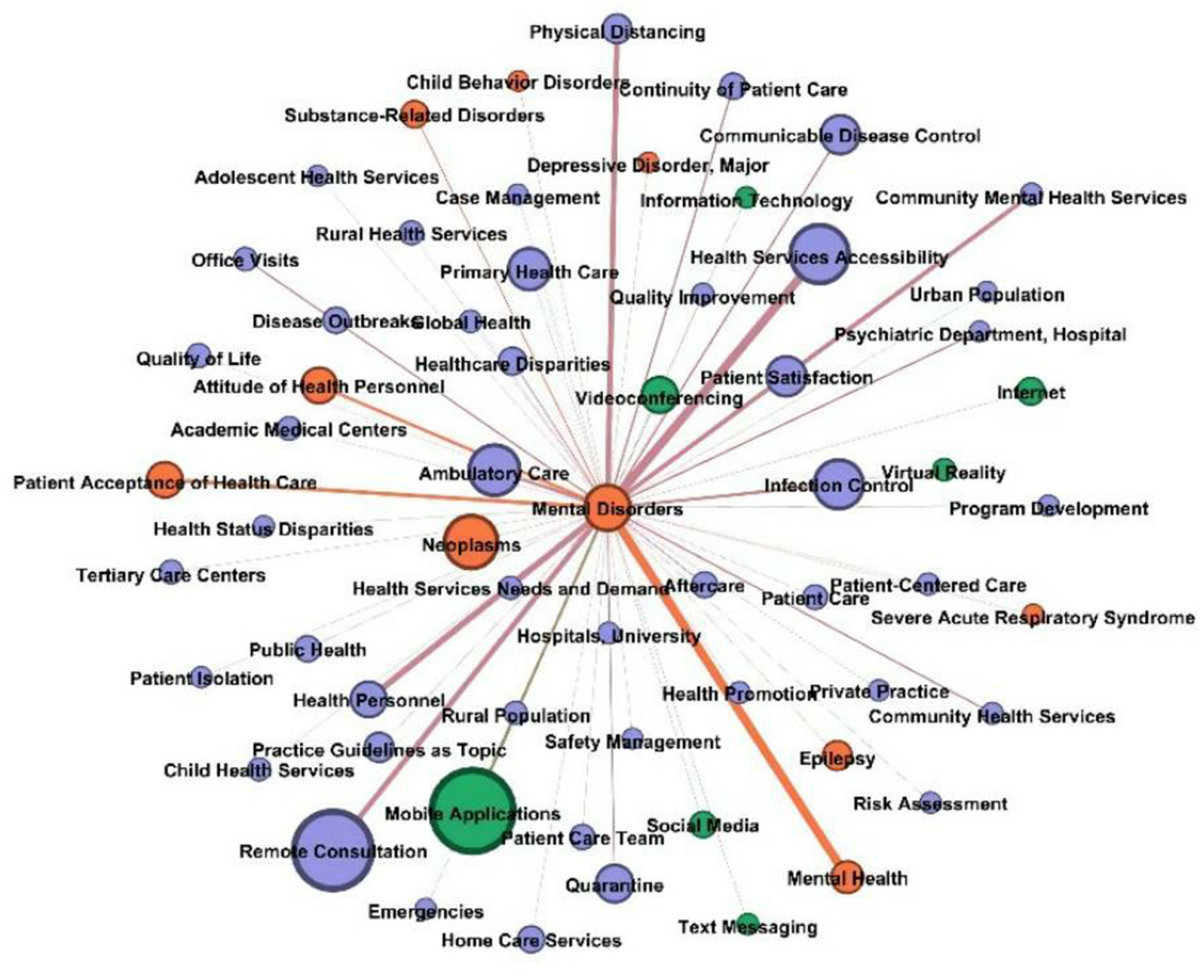
Mental disorders node and other nodes connections in the network.

Figure 7 shows the graphs of mental disorders. The health service entities associated with “mental disorders” were “health services accessibility,” “remote consultation,” “community mental health services,” and “health personnel.” In these networks, the significant disease entities were “substance-related disorders” and “attitude of health personnel.” The technology entities of “mobile applications” were frequently associated. Additionally, media entities were identified, such as text messaging, social media, the internet, videoconferencing, and virtual reality.

**Figure 8:**
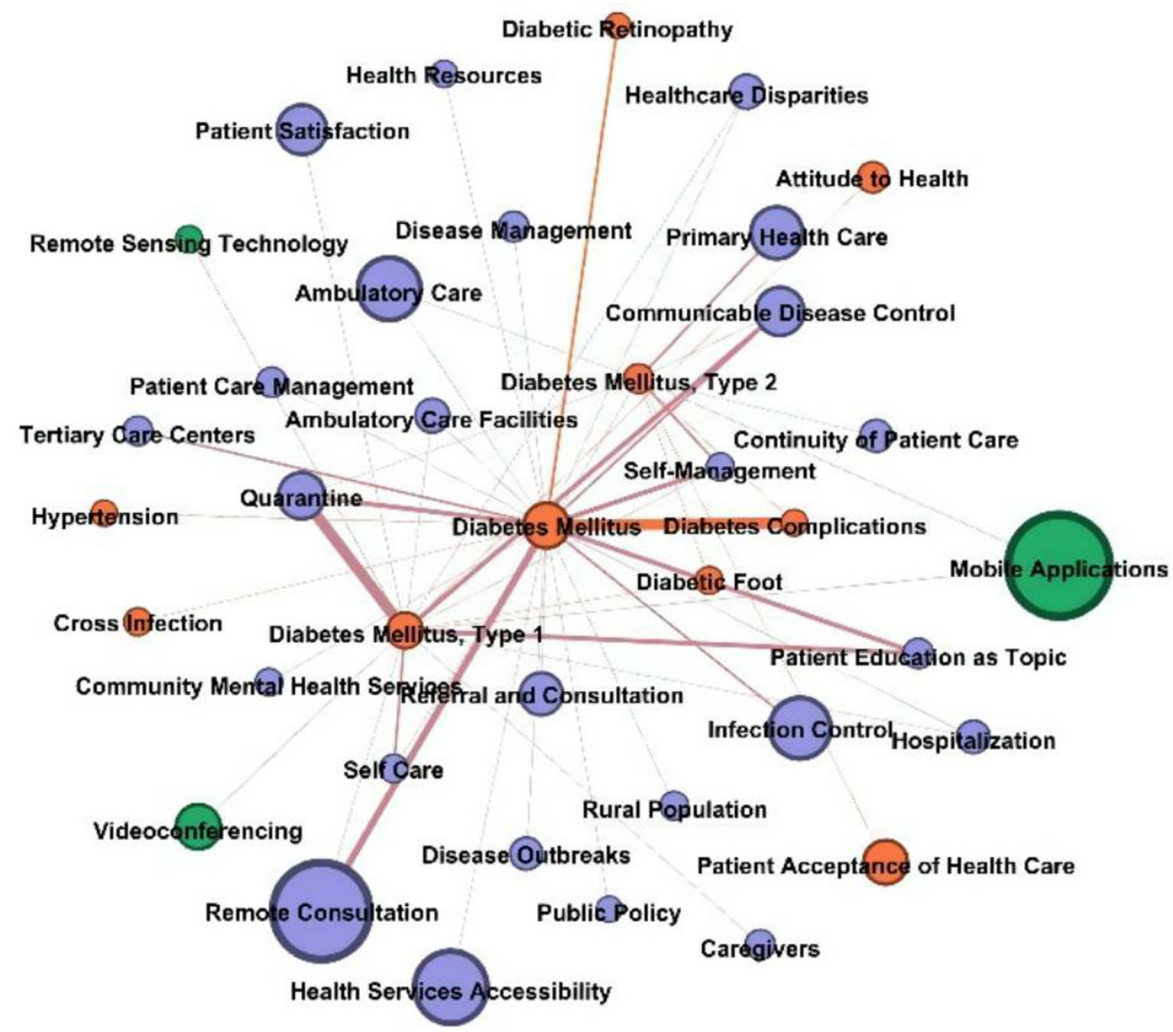
Diabetes mellitus node and other nodes connections in the network.

Figure 8 shows the graph of diabetes mellitus. The health service entities associated with “diabetes mellitus” were “remote consultation,” “quarantine,” “communicable disease control,” “patient education as topic,” and “self-management”. In these networks, the significant disease entities were “diabetes complications” and “diabetic retinopathy,” “diabetic foot”. The technology entities that jointed diabetes mellitus were “mobile applications,” “videoconferencing,” and “remote sensing technology”.

## Discussion

### Research Hotpots and Trends

Telemedicine has become increasingly popular during the COVID-19 epidemic. The results of bi-clustering analysis showed that telemedicine is mainly applied to provide mental health services, to delivery healthcare services, and to control cross-infection.

(1) Mental Health

Along with its high infectivity and mortality rates, COVID-19 has had a widespread emotional effect as a result of countrywide lockdowns and home confinement methods. Adverse psychosomatic effects in the general population are projected to rise dramatically as a result of the pandemic and the continual flow of easily accessible information and reinforced message gained *via* online social networking services. The public is much too bewildered, catastrophized, and morbidly concerned about the symptoms of COVID-19. The usual operation of healthcare systems may be disturbed in order to address widespread fear caused by widespread misrepresentation. These people include patients who tested positive for COVID-19, quarantined persons, health care staff, mental patients, and the general public. To address urgent and unmet psychological needs across several population domains during the COVID-19 pandemic, a novel psychosocial crisis prevention and intervention model should be established using suitable telemedicine technology (30). The most common forms of intervention include using smartphone APP [like PsyCovidApp (31), Insight Timer Meditation App (32)], videoconferencing (33, 34), internet (35, 36), and so on. Mobile applications (37), videoconferencing (38), text messaging (39), and social media (40) have shown to be useful communication methods for delivering mental health services.

(2) Delivery of Health Care Services

Telemedicine enables the assessment of a large number of patients while minimizing the danger of exposure to health care staff (41). Telemedicine is now employed in the following areas: primary care, illness management, medication monitoring, and rehabilitation.

- Primary Care: The epidemic of COVID-19 has altered the organizational structure and operations of primary care. Primary care services were recommended to employ 'virtual' consultations wherever feasible, which meant that each patient was required to have a telephone or video triage consultation to determine if an in-person visit was necessary. Numerous patients may be assessed at home through telemedicine or videoconferencing, preventing the transmission of infection to other patients and health care personnel. Telephone and videoconferencing are the most often utilized telemedicine technology in primary care (42). While the telephone is a known and trusted tool that is suitable for many discussions, the video may offer visual information, diagnostic hints, and therapeutic presence (43).
- Therapy: During the early stages of the COVID-19 worldwide pandemic, individuals with certain pre-existing medical issues were recognized as having an elevated risk, particularly those who need regular hospitalization for assessment and treatment. As is the case with patients suffering from chronic kidney disease, who need ongoing, life-saving renal replacement treatment and clinical monitoring (44). Infection control was promptly implemented in response to an increase in telemedicine usage among these patients. We may reduce physical congregating in institutions and preserve acceptable “social separation” *via* the use of telemedicine. Nephrologists and colleagues can guarantee that dialysis is safe and effective, while patients at home may “phone in” to telemedicine appointments and preserve the continuity of treatment (45). Furthermore, telemedicine technologies have been employed to treat cancer (46), skin diseases (47), eye diseases (48), and hypersensitivity (49), among other conditions.
- Drug Monitoring: Mobile health applications on smartphones support patients in medication self-management at home. These mobile health apps assist patients with self-management of medications by employing short-range data collecting capabilities or the camera on the phone to scan barcodes. David J Taber created a mHealth application to aid in the administration of drugs in kidney transplant patients. He discovered that the mHealth application aided in the improvement of self-monitoring and provider-driven medication management (50). Hubert Ebner proposed integrated medication management in mHealth applications that integrate Austrian electronic health record system ELGA eMedication and closed-loop mHealth-based telemonitoring to continuous medication monitoring of heart failure patients (51). To monitor dosage adjustments, F Nogueira developed a website to help monitor Antiphospholipid Syndrome patients, called Antiphospholipid Syndrome On Cloud or APSOnCloud (52).
- Rehabilitation: Physiotherapy is the most prevalent rehabilitation profession. Physiotherapy services assist individuals in developing, maintaining, and regaining their mobility and functional capacity, so enhancing their overall quality of life (53). As a result, many hospitalized COVID-19 patients in the acute phase and chronic COVID-19 patients need physical therapy treatments. Physiotherapists need in-person therapy, which increases the risk of coronavirus exposure. As a result, numerous tools and software for telerehabilitation have been devised and developed. For instance, Sword Health is a web-based tool that enables patients to conduct physiotherapy sessions at home while being watched in real-time and remotely by a team of physiotherapists and physicians who prescribe, assess, and oversee the whole procedure (54). This epidemic compelled physiotherapists to adapt to new digital technologies in order to continue practicing their profession, just as patients did in the digital age. Numerous Physical Therapists have opted for telemedicine in order to continue delivering therapy while minimizing in-person encounters and so limiting the virus's transmission. Videoconferencing was the most often utilized means of telerehabilitation, while other technologies such as ZOOM, telephone conversations, and facetime were also frequently used (55).

(3) Epidemic prevention

COVID-19 is difficult to detect using conventional techniques since patients are infectious for 1–2 days before developing symptoms and contacts become infected on average 3–4 days after exposure. Thus, the window for achieving containment by manual contact tracing is severely limited. Upon case confirmation, a mobile phone app may initiate contact tracing and notification. Keeping a brief database of close contacts of diagnosed patients might instantly inform them and motivate them to self-isolate. The application has been launched in China. Public health policy was accomplished *via* the use of an app that was optional but obligatory for movement between quarters, public places, and public transportation. The software enables the collection of data on user mobility and coronavirus diagnosis by a central database and displays a green, amber, or red code to indicate if movement restrictions should be relaxed or enforced (56). The program analyzes its database and assigns color codes using artificial intelligence. The app is a plug-in for WeChat and Alipay and has gained widespread use.

Ferretti et al. (57) has suggested an alternate strategy to curb the epidemic: app-based contact tracing. The concept is to leverage low-energy Bluetooth connections between phones to record user interactions, especially those that may represent a greater risk of infection. Another example of this kind of “privacy by design” COVID-19 tracing technique is the Singaporean government's TraceTogether app (58). In contrast to the contact point system, it needs merely consumers to activate Bluetooth on their phones. The European consortium's Pan-European Privacy-Preserving Proximity Tracing (PEPP-PT) standard (59), as well as Google and Apple's newly announced collaborative initiative (60), used a very similar approach.

Contact-tracking applications, on the other hand, need users to disclose sensitive information in order to work correctly, raising worries about data leakage, abuse, and social monitoring. With the risk that sensitive information would be leaked and utilized without their consent by harmful applications, consumers often take protective measures, such as deleting a mobile app.

### New Technologies in Telemedicine

From the results of social network analysis, we can see that mhealth applications are the most widely used telemedicine technology. Other technologies, like remote consultation and videoconferencing, were also prevalent telemedicine tools. However, some novel and advanced technologies have been used in certain fields, such as ophthalmology. In ophthalmology, artificial intelligence was utilized to screen diabetic retinas. Regulatory organizations acknowledge AI's promise in healthcare, and the FDA has cleared the use of an AI algorithm for the primary care diagnosis of diabetic retinopathy (61). The application of deep learning to ophthalmic images such as digital fundus photographs and visual fields has been shown to achieve high-precision automated screening and diagnosis of common vision-threatening diseases such as diabetic retinopathy (62), glaucoma (63), age-related macular degeneration (64), and retinopathy of prematurity (65).

According to the findings of the bi-clustering analysis, electronic health records and clinical decision support systems played an essential role during COVID-19. These telemedicine applications belong to medical informatics applications. They were automated systems applied to the patient care process, such as diagnosis, treatment, and means of transmitting medical data within the health care context. In the era of contemporary medicine, operational management of a pandemic requires utilizing the electronic health record (EHR), which may aid in the development of technologies to assist routine patient treatment (66). At the moment, commercial solutions such as the EPIC medical record system and NHS Attend Anywhere are available (67). Medical Informatics Applications can effectively support institutions during a pandemic by facilitating immediate widespread distribution of information, tracking transmission in real-time, creating virtual venues for meetings and day-to-day operations, and, perhaps most importantly, offering patients telemedicine visits. Worldwide, health institutions and government organizations resorted largely to medical informatics solutions in response to COVID-19. Clinical care was aided by the use of the EHR to educate frontline practitioners about new procedures, to give clinical decision assistance, and to enhance diagnostic testing systems.

### Barriers to the Application of Telemedicine

The use of telehealth is rapidly growing as technological infrastructure continues to evolve. During the COVID-19 pandemic, more health services are being delivered remotely. However, technical issues can obstruct data transmission and cause visual and hearing problems. Many individuals cannot benefit from telehealth due to various factors, such as the lack of proper infrastructure and low socioeconomic status. Nearly half of the world's population remains disconnected, despite the fact that 97 percent of the world's population resides within range of a cellphone signal (68). Additionally, not all internet connections are ideal for telemedicine, since internet capacity often varies by geographic area and internet subscription. Recent research (69) indicates that internet-based video consultations are more cost-efficient and successful in building rapport than telephone consultations. However, if other internet users hog the bandwidth, videoconference communication will be disturbed.

Second, only individuals with literacy skills can benefit from telemedicine (70). The likelihood of using telemedicine *via* teleconference is lower for those 65 years or older than for those 64 years or younger (71). The inability of older persons to use telehealth is mostly due to a lack of internet skills and technological acceptability. Although it is possible to perform telecare in some cases, people with visual or hearing impairments may require in-person visits. This can be challenging and adds to the complexity of a relationship between a healthcare provider and a patient (72).

Third, privacy (73) and confidentiality (74) were concerns for patients who used equipment in locations frequented by other family members, even more so when compulsory end-user agreements obliged patients to forfeit privacy and control over data. Commercialization of telemedicine services and extensive usage of mobile health applications and other devices increased privacy and cybersecurity risks. The American Medical Association's 2016 guidelines on physician ethics remain mandatory. Additionally, they provide principles for maintaining patient privacy and confidentiality, as well as delivering competent treatment (75).

### Limitations

This research completely and objectively reviewed the state of telemedicine progress during COVID-19 from a bibliometrics standpoint. We note, however, that our research has certain limitations. To begin, we searched just the PubMed database and did not examine the literature included in other databases, such as IEEE xplore. Second, this research covered a variety of different sorts of publications (letter, review, opinion, and original article). However, the articles used for bi-clustering analysis must have citation information that indicates how the kind of literature was filtered. Thirdly, we used MeSH keywords to extract technology, illness, and health care service entities. MeSH words, on the other hand, are mostly composed of commonly-used ideas, excluding new concepts. In future experiments, we will apply deep learning to extract entities from the complete text. Deep learning will be completely exploited to address the issue of named entity recognition in newly emerging terms and to alleviate the effort associated with human annotation.

## Conclusions

In our bibliometric study, we used visualization and text mining tools, as well as bi-clustering analysis, to investigate the trends, challenges, and technologies explored in the field of telemedicine during COVID-19. The results indicated that telemedicine had been widely used in COVID-19, and most studies relate to the delivery of health care and mental health services. The six clusters in this study provide an overview of telemedicine research on the following:

(1) telemedicine in the therapy of mental health during COVID-19; (2) the usefulness of mobile health devices in COVID-19 prevention and monitoring of close contacts; (3) telemedicine ensures patient access to care; (4) usage of telemedicine technology to prevent cross-infection and to monitor patient medication during isolation; (5) application of telemedicine technologies to treat chronic diseases; (6) The role of electronic health records and clinical decision support systems during COVID-19.

Additionally, entities for technologies, diseases, and health care services and their connections were illustrated in network graphs. The most frequently occurring entity in the network was the delivery of health care, followed by “remote consultation” and “infection control.” The most frequently occurring technology entity was mobile applications. Overall, our data showed that telemedicine during COVID-19 research focused on neoplasms, mental disorders, and diabetes. IT was primarily *via* mobile devices to deliver health care, remote consultation, control infection, and contact tracing.

Several implications follow from the findings from this effort to map the literature in telemedicine. Although some new technologies (AI, deep learning) have started to be used in telemedicine, they are only used in some disciplines, such as ophthalmology. Researchers in other disciplines may consider using these techniques in clinical diagnosis and treatment. Besides, Mobile devices have become the primary medium for telemedicine, especially in mental health services, and how to use them efficiently to provide more health services is still the focus of telemedicine research. Third, telemedicine tends to be used in different diseases. Physicians or researchers from different departments can refer to practices and applications from other departments.

This study aims to provide researchers with a comprehensive view of the various facets of telemedicine during COVID-19. It can help them identify critical trends and improve their understanding of the field. The study also highlighted the multiple applications of telemedicine in delivering health care while controlling infections. The study assists researchers in comprehending the knowledge structure in this sector, enabling them to discover critical topics and choose the best match for their survey work.

## Data Availability Statement

The original contributions presented in the study are included in the article/supplementary material, further inquiries can be directed to the corresponding author.

## Author Contributions

XL: data analyzing and writing initial draft and revision. HY: reviewing the literature and analyzing. LC: designing this study. All authors contributed to the article and approved the submitted version.

## Conflict of Interest

The authors declare that the research was conducted in the absence of any commercial or financial relationships that could be construed as a potential conflict of interest.

## Publisher's Note

All claims expressed in this article are solely those of the authors and do not necessarily represent those of their affiliated organizations, or those of the publisher, the editors and the reviewers. Any product that may be evaluated in this article, or claim that may be made by its manufacturer, is not guaranteed or endorsed by the publisher.
